# Real-Time Onboard 3D State Estimation of an Unmanned Aerial Vehicle in Multi-Environments Using Multi-Sensor Data Fusion

**DOI:** 10.3390/s20030919

**Published:** 2020-02-09

**Authors:** Hao Du, Wei Wang, Chaowen Xu, Ran Xiao, Changyin Sun

**Affiliations:** 1 School of Automation, Southeast University, Nanjing 210096, China; duhao@arist.ac.cn; 2 Institute of Applied Research Intelligent Science & Technology, Jiangsu and Chinese Academy of Sciences, Changzhou 213164, China; xuchaowen@arist.ac.cn (C.X.); xiaoran@arist.ac.cn (R.X.); 3 Jiangsu Collaborative Innovation Center of Atmospheric Environment and Equipment Technology (CICAEET), Nanjing University of Information Science & Technology, Nanjing 210044, China

**Keywords:** multi-sensor data fusion, multi-environments, state estimation, unmanned aerial vehicle

## Abstract

The question of how to estimate the state of an unmanned aerial vehicle (UAV) in real time in multi-environments remains a challenge. Although the global navigation satellite system (GNSS) has been widely applied, drones cannot perform position estimation when a GNSS signal is not available or the GNSS is disturbed. In this paper, the problem of state estimation in multi-environments is solved by employing an Extended Kalman Filter (EKF) algorithm to fuse the data from multiple heterogeneous sensors (MHS), including an inertial measurement unit (IMU), a magnetometer, a barometer, a GNSS receiver, an optical flow sensor (OFS), Light Detection and Ranging (LiDAR), and an RGB-D camera. Finally, the robustness and effectiveness of the multi-sensor data fusion system based on the EKF algorithm are verified by field flights in unstructured, indoor, outdoor, and indoor and outdoor transition scenarios.

## 1. Introduction

Multi-sensor data fusion (MSDF) is widely used in unmanned aerial vehicles (UAVs) due to the requirement of environmental adaptability, and it is at the core of sensing, estimation and perception in UAVs. An MSDF system can improve the ability of a UAV to adapt to a changeable and complex environment. In addition, MSDF technology is applied in fields such as autonomous driving, intelligent transportation, and medical diagnosis. Data for MSDF come from homogeneous or heterogeneous sensors. Generally, sensors are classified into two categories: interoceptive and exteroceptive [1]. Typical interoceptive sensors include gyroscopes, accelerometers, and wheel encoders. Exteroceptive sensors include visual sensors (e.g., RGB-D cameras, optical flow (OF) sensors, monocular cameras, stereo cameras, and fisheye cameras), Light Detection and Ranging (LiDAR), millimeter wave radar, and global navigation satellite system (GNSS) receivers (e.g., BeiDou Navigation Satellite System (BDS), Global Positioning System (GPS), and GLONASS). Inertial sensors, such as gyroscopes and accelerometers, have the advantages of concealment, autonomy, signal immunity, and information continuity, but are prone to accumulated errors. To date, simultaneous localization and mapping (SLAM) technology, including LiDAR SLAM and visual SLAM (vSLAM), has predominantly been used to solve the problem of autonomous navigation in a complex and unknown environment. LiDAR SLAM has the advantages of high precision and the ability to measure at long distances; its shortcomings include a high cost, its being greatly affected by rain and fog, and its providing less information on characteristics. The advantages of vSLAM are a low cost and an abundance of feature points; the disadvantages of vSLAM are its short-distance measurements, high computational requirements, and its susceptibility to environmental impacts. The navigation technology of the GNSS is relatively mature and often used in outdoor navigation. A single sensor cannot be used to solve the problem of state estimation in all environments because of the different disadvantages of interoceptive and exteroceptive sensors. In most cases, MSDF technology can effectively be used to solve the above problems. This technology can provide reasonable and good quality data [2]. By fusing the data from multiple sensors, we can not only expand the space of application of UAVs, but also improve the accuracy and reliability of state estimation for UAVs. In recent years, there have been several studies on state estimation based on multi-sensor fusion schemes [3,4,5,6,7] that can be applied to UAV or Unmanned Ground Vehicle (UGV) navigation. Some state estimation methods are only suitable for indoor environments, some are suitable for outdoor environments, and some are suitable for indoor and outdoor environments but are also used to estimate the position of UGVs. Other state estimation methods are suitable for three-dimensional (3D) UAV state estimation in indoor and outdoor environments. However, the fusion architecture is a centralized architecture and, since all operations are performed on one computer, once the equipment fails, the state estimation will fail. Therefore, the goal of this paper is to design a robust 3D pose estimation method, using multiple sensors, that can be applied to autonomous UAV navigation in multi-environments (indoor, outdoor and indoor-to-outdoor transition scenarios).

**Figure 1:**
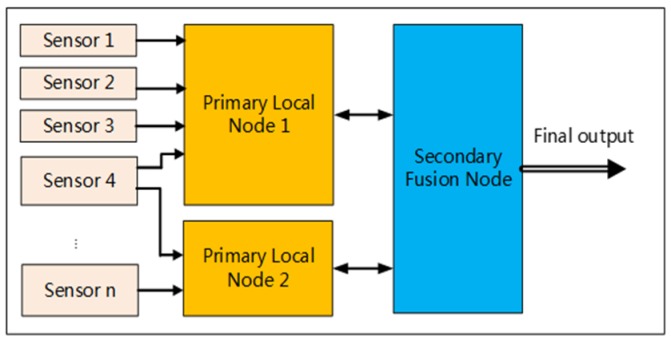
Hybrid fusion mode. The system consists of primary local nodes and a secondary fusion node.

In this paper, we focus on estimating the 3D state of a UAV in all environments, where a ‘state’ refers to the 3D position, the 3D attitude, and the 3D speed. The system employs an inertial measurement unit (IMU), the GNSS, an optical flow sensor (OFS), a depth camera, 3D LiDAR, and a barometer. The key innovations and contributions of this paper are:

(1) The 3D state is estimated by fusing data from multiple sensors (homogeneous or heterogeneous) in real-time, and can be applied to UAV navigation in multi-environments (indoor, outdoor, and outdoor GNSS-denied environments);
(2) In the fusion architecture, hybrid mode is chosen. First, the primary local nodes fuse some of the data from the sensors to obtain state information. Primary local node 1 is based on the data of IMU, magnetometer, GNSS, OFS and primary local node 2 is based on 3D LiDAR SLAM and vSLAM. Then, the secondary fusion node uses the Extended Kalman Filter (EKF) fusion algorithm to estimate the final state. Figure 1 shows the hybrid fusion architecture. In addition, we use a Controller Area Network (CAN) bus [8] interface to output UAV status information. CAN buses have priority and arbitration functions. Multiple modules are linked to the CAN bus through a CAN controller, which facilitates the increase or decrease in modules.

Next, we present related work. In Section 3, we describe the composition of the system. The EKF-based MSDF algorithm is presented in Section 4. We implement all of the ideas in our experimental platform and present the simulation and field experimental results in Section 5.

## 2. Related Work

It is well known that navigation, guidance, and control are relevant to robots [9]. Although control and guidance are very important, it is often necessary to first perceive the state of the robot.

Relevant work can be discussed in terms of sensor combination modes, application environments, and data fusion algorithms. Very few studies use a single sensor for 3D state estimation. Most of these studies employ the MSDF method in different scenarios, from which we can see that there are different limitations to the practical application of state estimation. The navigation technology of the GNSS/INS (Inertial Navigation System) is relatively mature and often used in outdoor navigation or situations where there are GNSS outages for a short time [10,11]. However, it is not suitable for indoor navigation or GNSS-denied environments. SLAM technology is currently the main way to perform navigation in unknown indoor environments [12]. Some of the recent literature has introduced state estimation using SLAM based on LiDAR/IMU [13,14,15,16], Camera/IMU [17,18,19], and LiDAR/Camera/IMU [20,21] for autonomous UAV navigation in indoor or GNSS-denied environments. In addition, the fusion of OFS and IMU is an important way to perform UAV state estimation in indoor environments [22]. However, a combination of these sensors can only be used in indoor environments. One of these combinations is famous and realizes real-time closed-loop detection based on Graph SLAM [23]; however, this method can only be applied to two-dimensional (2D) environments. 

In [3], the authors proposed a 3D state estimation algorithm for UAVs in unknown and GPS-denied environments. The algorithm uses an EKF to fuse the data from an IMU, a camera, and 2D LiDAR to achieve accurate positioning; however, this method cannot be applied to outdoor environments. In [4], an MSDF algorithm based on an Unscented Kalman Filter (UKF) is described, that integrates an IMU, LiDAR, stereo cameras, a GPS receiver, and other sensors. This approach can be applied to autonomous Rotor UAV flight in indoor and outdoor scenarios; however, the solution uses a centralized fusion method that is not convenient for system expansion. A good idea is to realize navigation in different environments by using the characteristics of different sensors [7]; however, this approach can only be used in UGVs. An MSDF algorithm based on a factor graph is proposed in [24], that can only be used in UAVs for autonomous outdoor flight. In [25], the authors achieved orientation and position estimation by complementary filter and Linear KF, however, this approach, which used GPS and a barometer, can only estimate the position outdoors. In [26], the authors present methods to fuse data from different sensors with a focus on attitude estimation algorithms, which solves the problems of autonomous control, state estimation, path planning, and remote operation, however, this method can only be used indoors. In [27], the authors proposed a multi-sensor-based autonomous outdoor navigation for UAV, a vision-based navigation system which provided pose observations in an EKF algorithm; this method can estimate the pose of the aircraft in real time. However, this system can only be used outdoors, and the maximum error of position is ± 5 m, while the maximum error of attitude is ± 3°. Various MSDF algorithms are described in [28], where the authors point out that the EKF and UKF methods can only deal with nonlinearity in a limited range. Nevertheless, as the selection of an appropriate algorithm depends on the application and the existing technology, in this paper, we focus on the use of an EKF algorithm to fuse the data from multiple sensors to solve the problem of navigation in multi-environments, which included an indoor scene, an outdoor scene, and indoor to outdoor transitions.

## 3. System Composition

The implementation of a fusion algorithm depends on a flight platform. This system consists of an IMU, a flight data recording (FDR), flight controller (FC), real-time kinematics (RTK), the GNSS, a three-axis magnetometer, MSDF, 3D LiDAR, an RGB-D camera, and an OFS. The IMU, the three-axis magnetometer, the FDR, the GNSS, and the flight controller constitute an independent flight control system (FCS).

**Figure 2:**
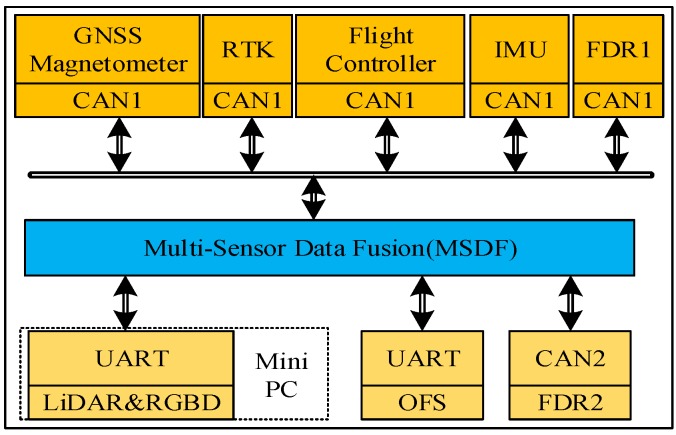
The modules of the system communicate in real time through the Controller Area Network (CAN) bus and the Universal Asynchronous Reciever/Transmitter (UART) bus.

In this paper, we adopt a hybrid fusion mode, including two-level fusion. The first-level local nodes perform local estimation of the UAV’s state and the second-level fusion node performs a global estimation of the UAV’s state. First-level fusion node 1 fuses the data from the IMU, magnetometer, GNSS, RTK, etc. This node can output the 3D position, attitude, and velocity of the UAV. An STM32F4 series processor is employed in first-level fusion node 1 as the operation unit. First-level fusion node 2 fuses the LiDAR, RGB-D camera, and IMU data and outputs the pose of the UAV. The second-level fusion node fuses the data from the two first-level nodes, and outputs the final pose and velocity of the UAV in multi-environments. The focus of this paper is the multi-sensor fusion module, i.e., the second-level fusion node. The fusion algorithm based on the EKF algorithm runs on the second-level fusion node. The computing platform of the second-level fusion node is an STM32F4 series processor. The IMU, FDR, FC, RTK, GNSS, and three-axis magnetometer sensors were connected to the MSDF algorithm via CAN bus 1, and the 3D LiDAR, RGB-D, and OFS sensors were connected to the MSDF algorithm via Universal Asynchronous Receiver/Transmitter (UART). FDR1 recorded the UAV’s flight data, and FDR2 recorded the fusion data. One of the advantages of using a CAN bus is that these sensors are networked together and can share data via the CAN bus. Another advantage of using a CAN bus is that sensors can be easily added and removed. A system composition diagram is shown in Figure 2.

In Figure 2, The GNSS, Magnetometer, RTK, Flight Controller, IMU and FDR1 are connected to the MSDF node via Controller Area Network (CAN) bus 1. FDR2 is connected to MSDF node through CAN bus 2; the computing unit used by LiDAR and RGB-D node is an X86-based CPU, and the SLAM algorithm based on LiDAR and vision runs on this processor; the LiDAR and RGB-D node connected to MSDF node through UART; the OFS module connected to MSDF node through another UART.

## 4. Multi-Sensor Fusion Algorithm

The Kalman filter (KF) [29] was created in the 1960s. After more than half a century of development, it remains one of the most powerful multi-sensor fusion algorithms for estimating the states of robots. The KF is generally applicable to state estimation in linear systems. However, many practical systems are nonlinear, such as the UAV system that we consider in this paper. Therefore, scholars have proposed many suboptimal approximate estimation methods, including the EKF, UKF, and particle filter (PF). In view of our hardware conditions and previous design experience, we selected the EKF as the data fusion algorithm in this paper.

### 4.1. MSDF System Model

In this study, the sensors were directly fixed onto the UAV, and were pre-corrected. The model of the continuous-time nonlinear system of the UAV based on the EKF for MSDF is expressed as follows

(1) $$x_{1}=f\left(x_{1},u\right)+G_{1}w_{1}$$

where $x_{1}={\left[\begin{array}{cccccc} p & v & \bar{q} & b_{gyro}^{b} & b_{a}^{b} & b \end{array}\right]}^{T}\in R^{17\times 1}$ denotes the state of the MSDF system,$p={\left[\begin{array}{ccc} p_{x} & p_{y} & p_{z} \end{array}\right]}^{T}$ denotes the position with respect to the world frame, $v={\left[\begin{array}{ccc} v_{x} & v_{y} & v_{z} \end{array}\right]}^{T}$ is the 3D velocity in the North East Down (NED) frame, $\bar{q}={\left[\begin{array}{cccc} q_{1} & q_{2} & q_{3} & q_{4} \end{array}\right]}^{T}$ are the quaternions in the world frame, which are used to represent the attitude of the UAV, $b_{gyro}^{b}$, $b_{a}^{b}$ represent the bias of the gyroscopes and accelerometers, respectively, in the body frame, and $b_{h}$ is the altitude bias of the 3D LiDAR, single-line Laser range finder, or barometer in the world frame. In primary local node 1, the altitude error that is estimated based on LiDAR SLAM is large, and the altitude error of the barometer is also large. Therefore, the altitude error $b_{h}$ was added to the system state equation as a state variable in order to improve the accuracy of height estimations. The system equation does not take into account the control input u. We assume that $w_{1}$ is the zero-mean Gaussian process noise, $w_{1}∼N(0,Q)$.

#### 4.1.1. The State Equations of the MSDF System

We can obtain the differential equations of the MSDF system based on [3,30,31].

(2) $$\dot{p}=v$$

(3) $$\dot{v}=C(\bar{q})\left(a_{m}-b_{a}\right)+{\left[\begin{array}{ccc} 0 & 0 & g \end{array}\right]}^{T}$$

(4) $$\dot{\bar{q}}=\frac{1}{2}\bar{q}⊗(w_{m}-b_{gyro})$$

(5) $${\dot{b}}_{gyro}=0,{\dot{b}}_{a}=0,{\dot{b}}_{h}=0$$

In Equation (3), $C\left(\bar{q}\right)$ denotes a rotational matrix. In Equation (4), $\omega_{m}$ denotes the angular velocity, which can be obtained from the gyroscopes. We can obtain Equation (5) based on Equations (15) and (18), which are described in Appendix A.

#### 4.1.2. Relative Measurement Model

The measurement model of the MSDF system in an indoor environment contains three sensor units: the LiDAR module, the RGB-D module, and the OF module. Each sensor module is independent and can be considered as a black box. The LiDAR module that was used in this study was a 3D scanner able to output 3D position and attitude estimations in space. The RGB-D module can output a 3D estimation of pose. The OF module can output the velocity in the X and Y directions. However, the altitude estimations from the LiDAR and the RGB-D modules are insufficiently accurate, and neither sensor module outputs 3D velocity estimations. Although the OF sensor module has a 2D velocity output, it is typically used when a UAV is hovering and is not suitable for large-scale flight operations. In order to solve these problems, we estimated the state of the UAV in multi-environments by constructing an EKF model based on a variety of homogeneous and heterogeneous sensors. Based on the above, the observation equation is given as follows

(6) $$y_{1}=h(x_{1})+v_{1}$$

where $y_{1}={\left[\begin{array}{ccc} p & v & a \end{array}\right]}^{T}\in R^{9\times 1}$ comprises the position measurement p, the velocity measurement $v^{b}$, and the acceleration measurement a. p comes from the LiDAR module or the RGB-D module, a comes from the IMU, $v={\left[\begin{array}{ccc} v_{x} & v_{y} & 0 \end{array}\right]}^{T}$ comes from the OF module, and $v_{1}$ represents the zero-mean Gaussian measurement noise, $v_{1}∼N(0,R_{1})$.

#### 4.1.3. Extended Kalman Filter Algorithm

In field engineering applications, the functions f(·) and h(·) are usually nonlinear and need to be linearized. The state transition matrices $F_{k}$ and $H_{k}$ can be obtained by calculating partial derivatives of the nonlinear function, that is, calculating the Jacobian matrix of these functions, as follows

(7) $$F_{k}={\frac{\partial f}{\partial x}|}_{x={\hat{x}}_{k-1}},H_{k}={\frac{\partial h}{\partial x}|}_{x={\hat{x}}_{k}}$$

where ${\hat{x}}_{k}$ is the estimate of $x_{k}$, and we can obtain $F_{k}$ and $H_{k}$ after derivation, respectively.

$$F_{k}=\left[\begin{array}{cccccc} 0_{3\times 3} & I_{3\times 3} & 0_{3\times 4} & 0_{3\times 3} & 0_{3\times 3} & 0\\ 0_{3\times 3} & 0_{3\times 3} & \delta 1 & 0_{3\times 3} & -C{\left(\bar{q}\right)}_{3\times 3} & 0\\ 0_{4\times 3} & 0_{4\times 3} & \delta 2 & \delta 3 & 0_{4\times 3} & 0\\ 0_{3\times 3} & 0_{3\times 3} & 0_{3\times 4} & I_{3\times 3} & 0_{3\times 3} & 0\\ 0_{3\times 3} & 0_{3\times 3} & 0_{3\times 4} & 0_{3\times 3} & I_{3\times 3} & 0\\ 0_{1\times 3} & 0_{1\times 3} & 0_{1\times 4} & 0_{1\times 3} & 0_{1\times 3} & 1 \end{array}\right]$$

Here, $\delta_{1}=(a_{m}-b_{a})\frac{\partial (C\left(\bar{q}\right))}{\partial \bar{q}}$, $\delta_{2}=\frac{\partial f}{\partial \bar{q}}$, $\delta_{3}=\frac{\partial f}{\partial b_{gyro}}$.

$$H_{k}=\left[\begin{array}{cccc} I_{3\times 3} & 0_{3\times 3} & 0_{3\times 4} & 0_{3\times 7}\\ 0_{3\times 3} & I_{3\times 3} & 0_{3\times 4} & 0_{3\times 7}\\ 0_{3\times 3} & 0_{3\times 3} & \delta 3_{3\times 4} & 0_{3\times 7} \end{array}\right]$$

Here, $\delta 3={\frac{\partial h}{\partial \bar{q}}|}_{3\times 4}$.

Based on the above parameters, we can use the well-known EKF to estimate the pose and velocity of a UAV. The procedure is as follows.

EKF Algorithm ($x_{k-1},P_{k-1},y_{k}$):

(8) $$x_{k|k-1}=F_{k|k-1}x_{k-1}$$

(9) $$P_{k|k-1}=F_{k|k-1}P_{k-1}F_{k|k-1}^{T}+Q_{k-1}$$

(10) $$K_{k}=P_{k|k-1}H_{k}^{T}{\left[H_{k}P_{k|k-1}H_{k}^{T}+R_{k}\right]}^{-1}$$

(11) $$x_{k}=x_{k|k-1}+K_{k}\left[y_{k}-H_{k}x_{k|k-1}\right]$$

(12) $$P_{k}=\left[I-K_{k}H_{k}\right]P_{k|k-1}$$

return $x_{k},P_{k}$.

In this study, both $Q_{k}$ and $R_{k}$ were set to be constant diagonal matrices. As mentioned above, state estimation in an indoor environment is usually based on LiDAR or a visual sensor, and the measured value is a relative measurement [4].

#### 4.1.4. Absolute Measurement

In an outdoor environment, the GNSS receiver can be used to estimate the state of a UAV, as it has the ability to provide absolute measurements. Because the measurement models of the two systems are different, the absolute measurement model is introduced separately. The details are as follows

(13) $$y_{2}={\left[\begin{array}{cc} p^{LLA} & v^{r} \end{array}\right]}^{T}+v_{2}$$

where $y_{2}$ is the observed measurement from the GNSS receiver, $p^{LLA}={\left[\begin{array}{ccc} \phi & \lambda & h \end{array}\right]}^{T}$ represent the latitude, longitude, and altitude, respectively, in the Latitude, Longitude, Altitude (LLA) coordinate frame, $v^{r}={\left[\begin{array}{ccc} v^{N} & v^{E} & v^{D} \end{array}\right]}^{T}$ denote the velocity in the North East Down (NED) frame, and $v_{2}∼N(0,R_{2})$ denotes the absolute measurement noise.

## 5. Simulation and Experiment

Before a field experiment, a simulation is necessary to verify whether a system model is correct. The key parameters of an MSDF system, such as the covariance matrices Q and R, are often adjusted through simulation. The MSDF system was simulated in MATLAB.

### 5.1. Simulation

The pose and velocity of primary node and high-precision sensor data were collected by UAV before field experiments, and the sensors involved in this study were mounted on a drone to obtain simulation data through an actual flight. The fusion algorithm was run in MATLAB, and the offline fusion state data were compared with the data of high-precision sensors to test the effectiveness of the MSDF algorithm. Next, we introduced the performance of the commercial high-precision sensors (IMU inertial sensors, RTK systems, and a 3D motion capture system (VICON)) that we used to compare the accuracies of pose estimations. The high-precision IMU sensor that we used was Ellipse-N, which came from SBG Systems [32]. VICON is a highly accurate system that is the premier solution for drone studies, providing a ground truth for UAV localization experiments. The details of their performance are shown in Table 1.

**Figure 3:**
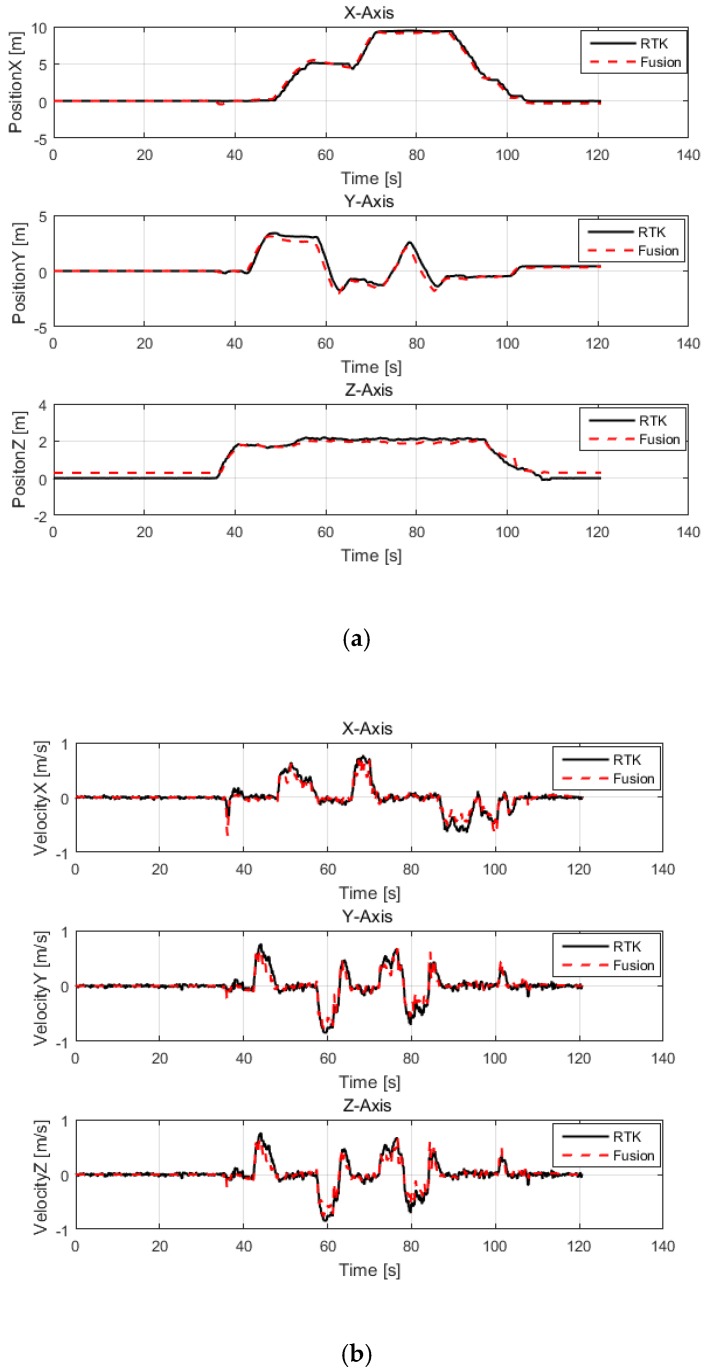

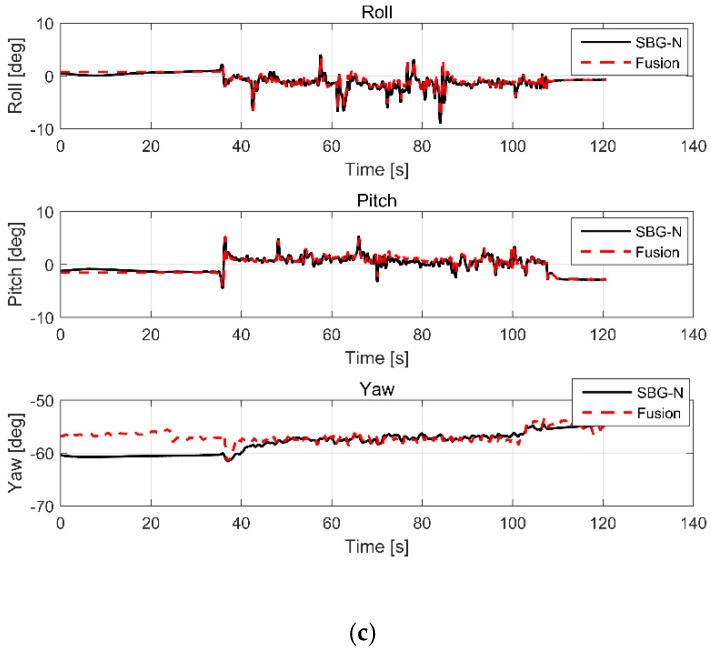
Comparison of multi-sensor data fusion (MSDF)-based estimation and high-precision sensors in an outdoor environment. (**a**) Contrast between the position estimations of the real-time kinematics (RTK) and fusion-based systems; (**b**) Contrast between the velocity estimations of the RTK and fusion-based systems; (**c**) Contrast between the attitude estimations of the IMU (SBG-N) and fusion-based systems. The red line represents the fusion data and the black line represents the ground truth, which was provided by the RTK system or the high-precision inertial measurement unit (IMU).

The simulation was based on the above MSDF system model, and included position, velocity and attitude. In order to verify the accuracy of pose and velocity estimations, we compared our results with the data on high-precision IMU, RTK, and VICON sensors; the experimental data for the simulation were taken from the data collected by a UAV during an actual flight, and the scenes included an indoor scene, an outdoor scene, and indoor to outdoor transitions. The outdoor scene position accuracy was compared with the RTK system, and the indoor scene position accuracy was compared with the VICON system. In an outdoor environment, because the LiDAR SLAM algorithm only works when there are reflections around 3D LiDAR, we collected data in an outdoor environment that contained obstacles. The results of the comparison of different states are shown in Figure 3.

In order to evaluate the accuracy of the fusion algorithm in an outdoor environment, the sensors involved in the MSDF system and the high-precision sensors that were used for comparison were simultaneously mounted onto the UAV, and the ground truth was provided by the RTK system in the outdoor environment. Then, we manually flew the UAV in attitude mode and recorded data on the UAV’s state in the FDR. The state value that was estimated by the fusion algorithm was compared with the value estimated by the high-precision sensor in MATLAB. In addition, we converted the state values from different coordinate systems to the same coordinate system. As can be seen from Figure 3a,b, the position and velocity estimated by the fusion algorithm follow the position and velocity estimated by the RTK system, and the maximum position error accuracy is less than 10 cm. As shown in Figure 3c, the maximum error of ± 3° occurred after the flight stabilized, which meets the flight requirements of the rotorcraft.

**Figure 4:**
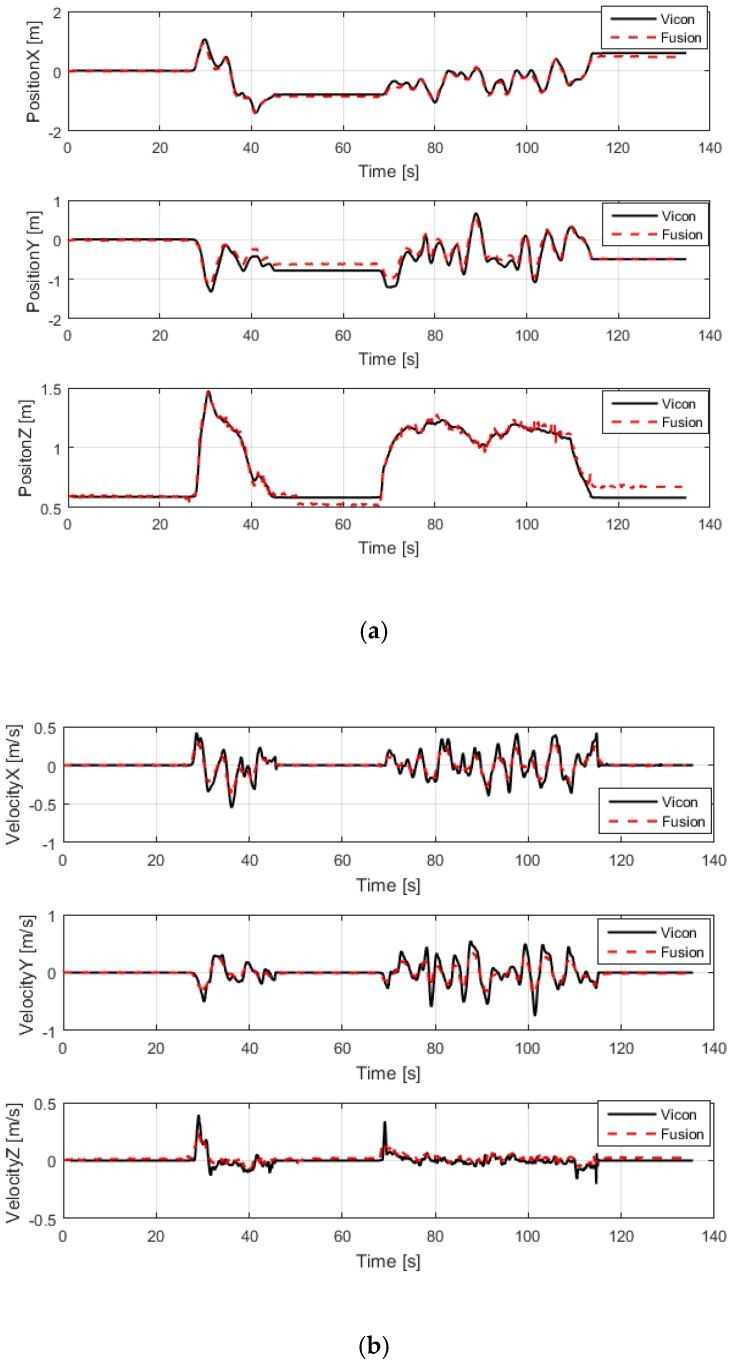

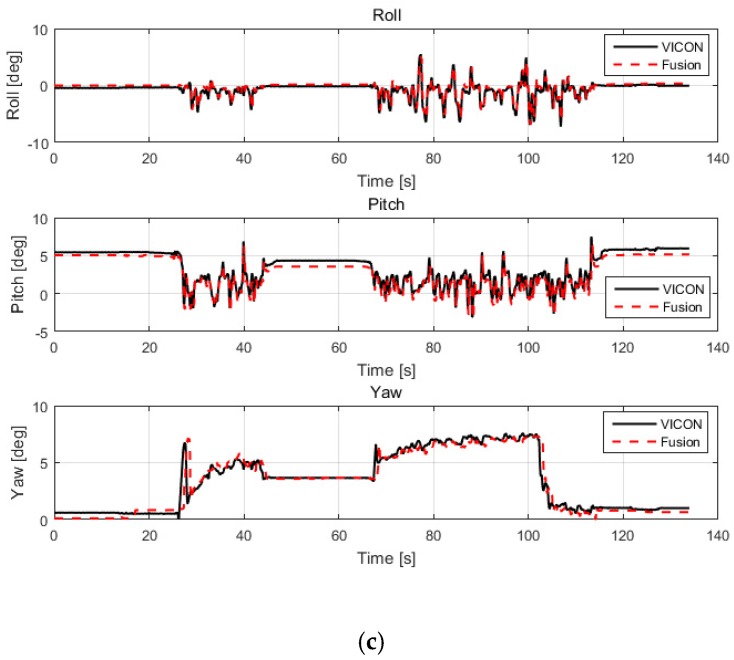
Comparison of the estimated pose and velocity of the unmanned aerial vehicle (UAV) in an indoor environment. (**a**) Contrast between the position estimation of the VICON system and that of the fusion-based system; (**b**) contrast between the velocity estimation of the VICON system and that of the fusion-based system; (**c**) contrast between the attitude estimation of the VICON system and that of the fusion-based system. The red line represents the fusion-based system’s velocity estimation, and the black line represents the ground truth, which was provided by the VICON system.

In the indoor environment, the estimated pose and velocity were compared with a ground truth that was provided by the VICON system. The results of the comparison are shown in Figure 4.

In the indoor environment, we evaluated the accuracy of the fusion algorithm’s state estimation by comparing it with the ground truth provided by the VICON system. First of all, we sent the data on the pose and velocity of the UAV to a computer through a wireless link, and the computer also received the data provided by the VICON system, so that the accuracy of the fusion algorithm could be verified by comparing the data, and then we manually flew the UAV in attitude mode and recorded data on the UAV’s state in the personal computer (PC). The state value that was estimated by the fusion algorithm was compared with that estimated by the VICON system in MATLAB. As can be seen from Figure 4a,b, the position and velocity estimated by the fusion algorithm follow the velocity and position estimated by the VICON system, and the maximum position error accuracy is less than 10 cm. As shown in Figure 4c, there is a maximum error of ± 3°, which meets the flight requirements of the rotorcraft. Next, we show the state estimation in an indoor-to-outdoor transition area.

**Figure 5:**
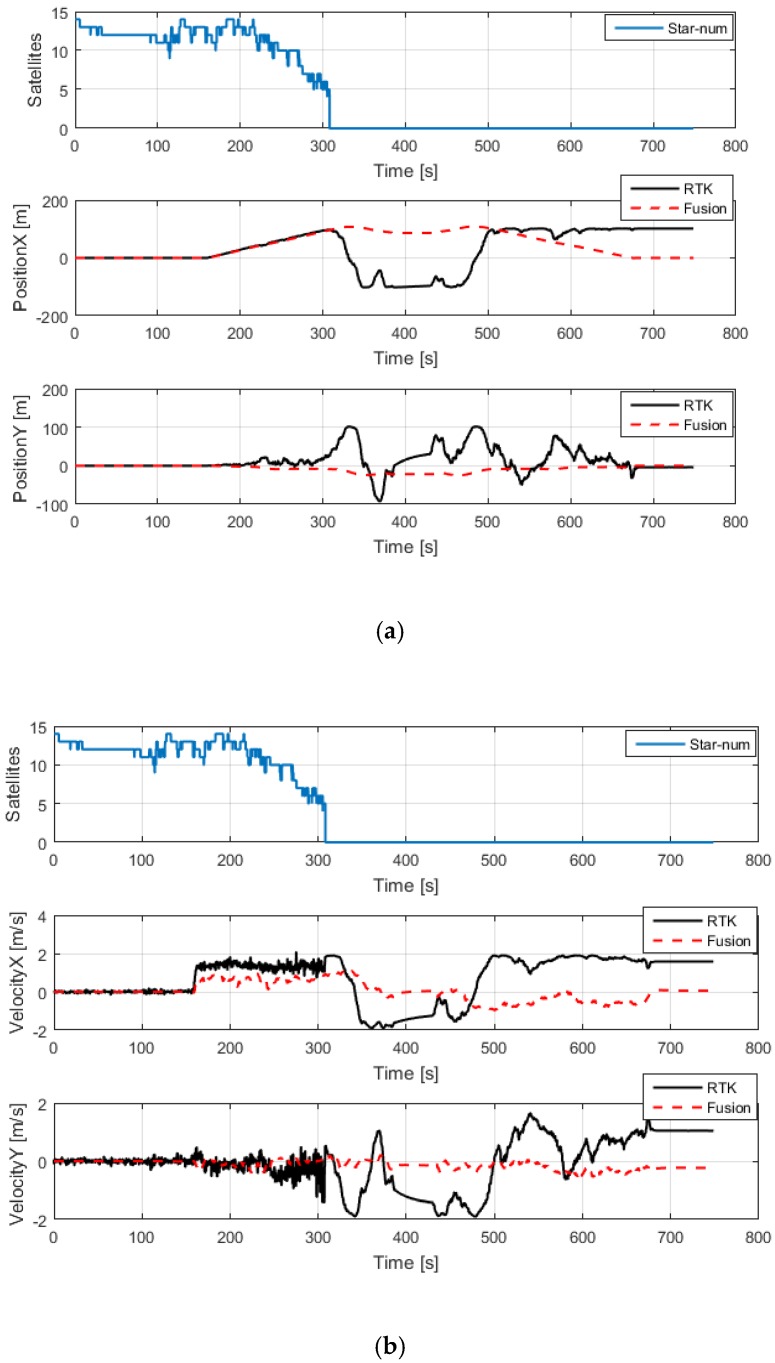
Comparison of the state estimation of the RTK system and the fusion-based system in an indoor-to-outdoor transition area. (**a**) The number of satellites and the comparison curve for position; (**b**) the number of satellites and the comparison curve for velocity. The blue solid line represents the number of satellites, the red solid line represents the fusion data, and the black solid line represents the ground truth, which was provided by the RTK system.

It can be seen from Figure 5 that the satellite signal is good between 0 and 300 s. The position estimated by the fusion algorithm is consistent with the position estimated by the RTK system. After 300 s, there is no satellite positioning signal, because the drone transitioned from outdoors to indoors; the position and velocity estimated by the multi-sensor fusion algorithm remained normal, and the position and velocity estimated by the RTK system began to drift. After 700 s, the position and velocity estimated by the multi-sensor fusion algorithm returned to their original values.

### 5.2. Field Experiment

In this section, we introduce the UAV platform, the power allocation, and the state estimation results for different experimental scenarios.

#### 5.2.1. Experimental Platform

**Figure 6:**
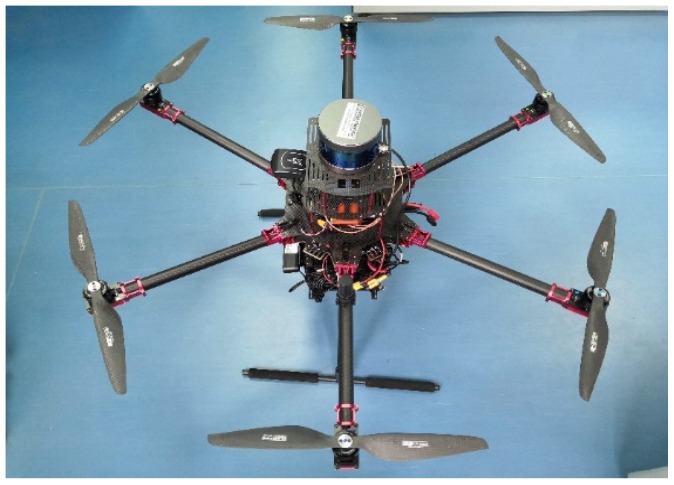
Our 8.5 kg drone platform (including paddles and batteries), which is equipped with three-dimensional (3D) Light Detection and Ranging (LiDAR), an RGB-D camera, an inertial measurement unit (IMU), an optical flow sensor (OFS), a barometer, and a global navigation satellite system (GNSS) receivers (BDS/GPS, RTK).

In addition to the simulation, we also verified the effectiveness of the algorithm through a practical experiment. The sensors that we used in the experimental platform that we employed in this study included 3D LiDAR, RGB-D, IMU, OF, barometer, and GNSS receiver (BDS/GPS and RTK) sensors. The computing unit was equipped with two embedded boards and an X86-based PC. Our UAV flight platform is shown in Figure 6. The main specifications of the flight platform are shown in Table 2.

#### 5.2.2. Introduction to the Calculation of Power

The MSDF system introduced in this paper contains first-level local fusion nodes and a second-level global fusion node. The first-level local fusion nodes are independent. The local fusion nodes include the IMU and the LiDAR and RGB-D modules. Attitude estimation using data from the IMU is performed by an embedded STM32F4 series processor, which can output an attitude estimation or the quaternion at 50 Hz. The 3D LiDAR module can output a complete 3D pose estimation at 20 Hz. The RGB-D module updates more slowly than the LiDAR module, and can output a complete 3D pose estimation at 10 Hz. The Graph-SLAM algorithm running on the LiDAR and RGB-D modules is based on a Robot Operating System (ROS) and the hardware platform is based on mini PC (i5-8250u), with a frequency of 3.4 GHz and 16 GHz of RAM. The second-level global fusion node is used to process the data from the first-level local fusion nodes. Its computing platform is a STM32F405 series processor with a 168 MHz CPU, 210 DMIPS, and 1 Mb of Flash memory. It can output complete 3D position and velocity estimations at 10 Hz and attitude estimations at 50 Hz.

#### 5.2.3. Experimental Results

In order to prove that our MSDF system can be applied in all environments, we chose an indoor environment, a woodland area, an area near high-rise buildings, and an indoor–outdoor transition area to verify the effectiveness of the MSDF system. We provide the experimental results that were obtained in the area near the high-rise buildings.

**Figure 7:**
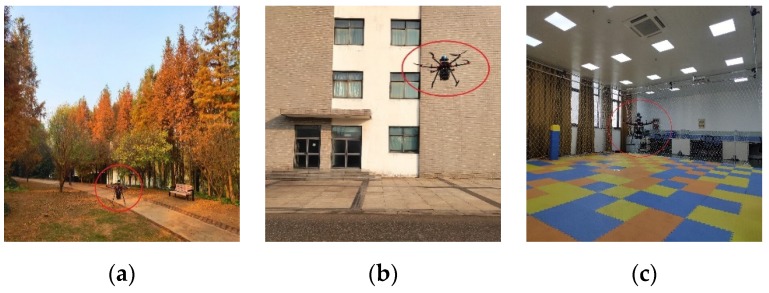
Images of the UAV in flight in different environments. (**a**) The drone flying in the woods; (**b**) The drone flying adjacent to high-rise buildings; (**c**) The drone flying in an indoor environment.

The drone can only achieve hover control and velocity control based on the fused data of MSDF system; the experimental results are shown in Figure 7a–c. In Figure 7a, the hover scene was a wood. In Figure 7b, the drone was hovering adjacent to high-rise buildings, and in Figure 7c, the drone was hovering in an indoor environment.

**Figure 8:**
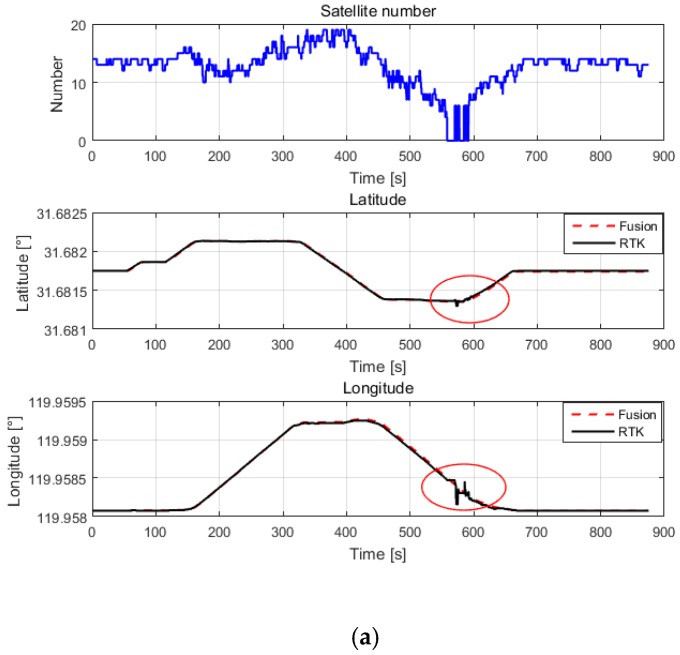

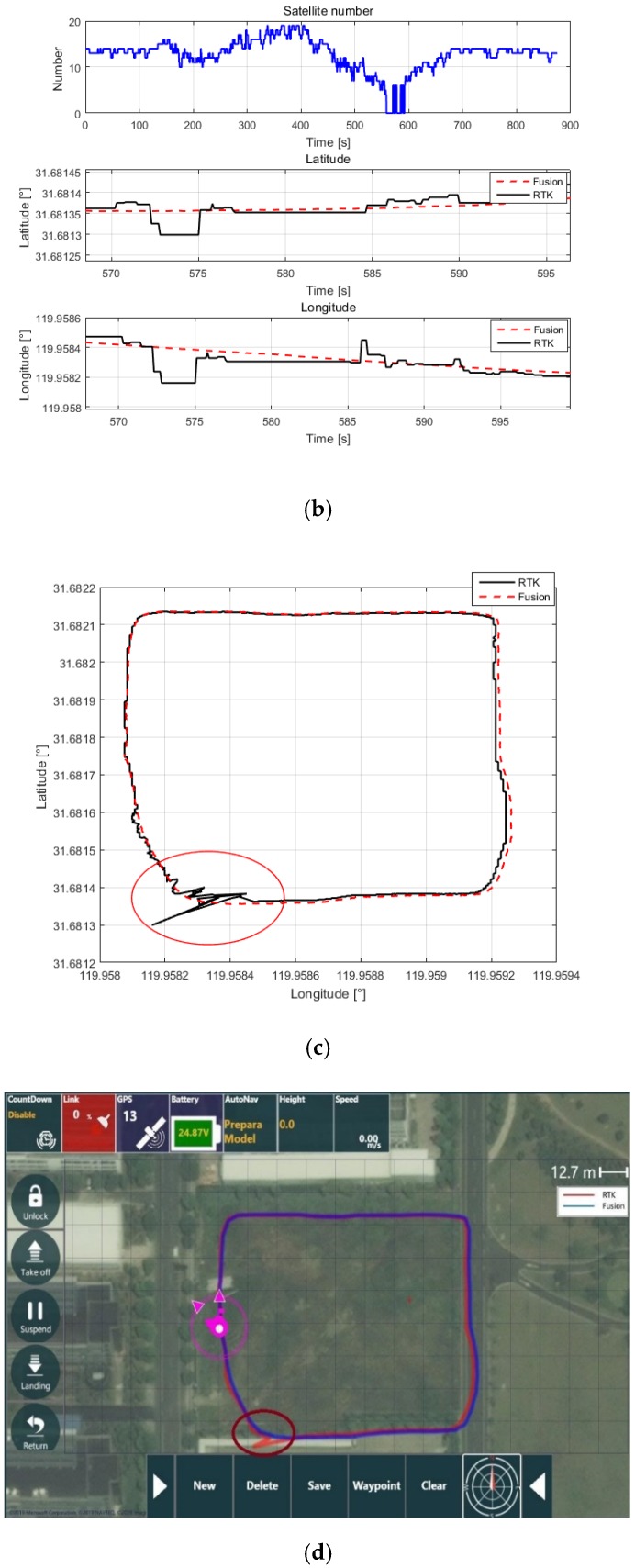
The flight trajectory of the UAV near high-rise buildings. (**a**–**c**) A comparison of the traces displayed in MATLAB. The red dashed line represents data from the fusion-based system, and the black line represents the ground truth, which was provided by the RTK system; (**d**) A comparison of the trajectories of the UAV via the station on the ground. The blue line represents the data from the fusion-based system, and the red line represents the ground truth provided by the RTK system.

We used the manual attitude mode to fly the UAV adjacent to high-rise buildings. The experimental results are shown in Figure 8, and were transformed by coordinates and units. The data shown in Figure 8a–d were calculated in real time through the embedded board (the details are described in Section 3 and Section 5.2.2). These data were stored in the FDR. The trajectory was obtained by simultaneously transmitting the position data from the onboard RTK system and the position data from the embedded board to a station on the ground through a wireless link. The number of satellites is shown at the top of Figure 8a. The area that is marked by red ellipses in Figure 8a shows the trajectory when the number of satellites is less than six, and is enlarged, as shown in Figure 8b. It can be seen that the position data from the onboard RTK system, which depend on a GNSS signal, starts to drift and jump, while the position estimated by the fusion algorithm remains stable. In Figure 8c, the horizontal axis represents longitude and the vertical axis represents latitude. The data shown in Figure 8d were obtained by sending the position of RTK and MSDF to the ground station computer at the same time, the red line represents the position of the RTK, and the blue line represents the fused position. The areas that were marked by red ellipses in Figure 8c,d show the stage of drift from another perspective.

## 6. Conclusions

In this paper, a hybrid MSDF architecture was presented. The first-level local fusion nodes in the proposed MSDF architecture are regarded as black boxes that are independent of each other and connected by a CAN bus or a UART bus. The most important advantage of this architecture is that local sensor fusion nodes can be conveniently added or removed according to a task’s requirements. The second-level global node fuses the results from the first-level local fusion nodes using the EKF algorithm. The convergence of the algorithm was verified by a simulation, and the covariance matrices Q and R were optimized and adjusted. Then, the real-time performance and practicability of the EKF algorithm were verified by experiments in indoor, forest, a high-rise building vicinity, and indoor–outdoor transition areas. From the simulation and experimental results, it can be seen that the proposed MSDF system not only estimates states that a single sensor is unable to observe, but also enhances the space coverage and improves the accuracy of the estimated values of the state variables. The sensor fusion method proposed in this paper provides target-level fusion. In the future, we will try to fuse data from the raw data layer and adopt intelligent algorithms, such as deep learning, to achieve MSDF. In addition, with the development of solid-state LiDAR towards light-weight and low-cost sensors, the application of 3D LiDAR in Rotor UAVs will become more widespread.

## Figures and Tables

**Table 1:** Comparison of the accuracy of different sensors.

Sensor	State	Accuracy
IMU ELLIPSE-N	Roll/Pitch Heading	0.1° 0.5°
GNSS (RTK)	Horizontal Position	1 cm + 1 ppm
GNSS (RTK)	Vertical position	2 cm + 1 ppm
GNSS (RTK)	Velocity	<0.03 m/s
VICON	Position	<0.5 mm

**Table 2:** UAV Platform specifications.

Type	SPECS
Weight (with 12,000 mAh TATTU batteries)	8.5 kg
Diagonal Wheelbase	1000 mm
Max Takeoff Weight	12 kg
Hovering Accuracy (RTK)	Vertical: ± 10 cm, Horizontal: ± 10 cm
Max Speed	43 km/h (no wind)
Max Wind Resistance	10 m/s
Hovering Time	No payload: 25 min, 3 kg payload: 10 min

## Appendix A

The inertial sensors that we used in this study are based on the Micro Electro Mechanical System (MEMS) technology, and are inaccurate in long-term operation. Measurements of the MEMS accelerometer and gyroscope are denoted as [33].

(A1) $$a_{m}=a+b_{a}+n_{a},w_{m}=w+b_{gyro}+n_{gyro}$$

where $a,\omega \in R^{3}$ denotes the real value, $b_{a},b_{gyro}\in R^{3}$ denotes the bias, $n_{a},n_{g\mathrm{yro}}\in R^{3}$ denotes the white Gaussian noise, and $E\left(n_{a}\right)=0_{3\times 1},E(n_{gyro})=0_{3\times 1}$. Furthermore, the bias $b_{a},b_{gyro}$ is modeled as follows

(A2) $${\dot{b}}_{a}=n_{b_{a}},{\dot{b}}_{gyro}=n_{b_{gyro}}$$

Single-line laser range finders and barometers are used to measure the relative and absolute altitudes of drones. Laser range finders are also used to measure relative height, and are often mounted on the bottom of a drone and face downward. The measured altitude is denoted by $d_{Laser}$:

(A3) $$d_{Laser}=\frac{-1}{\cos\theta \cos\varphi }p_{h_{e}}+n_{d_{Laser}}$$

where θ,φ denote the pitch and roll angle, respectively, $-p_{z_{e}}$ denotes the real height from the ground, and $n_{d_{Laser}}$ denotes the white Gaussian noise in the single-line laser range finder.

Barometers are commonly used to measure the absolute and relative altitudes of drones. The altitude $d_{b}$ is denoted as

(A4) $$d_{b}=-p_{h_{e}}+b_{d_{b}}+n_{d_{b}}$$

where $b_{d_{b}}$ denotes the bias and $n_{d_{b}}$ represents white Gaussian noise. $b_{d_{b}}$ can be expressed as

(A5) $${\dot{b}}_{d_{b}}=n_{b_{d_{b}}}$$

where $n_{b_{d_{b}}}$ represents the white Gaussian noise in the barometer. Next, we discuss the multi-sensor fusion navigation scheme in indoor and outdoor scenarios.
